# Microstructural Investigation of LME Crack Initiated in Ferritic/Martensitic Steel T91 Loaded in Liquid Lead-Bismuth Eutectic at 300 °C

**DOI:** 10.3390/ma12010038

**Published:** 2018-12-22

**Authors:** Patricie Halodová, Jan Lorinčík, Anna Hojná

**Affiliations:** Centrum Výzkumu Řež (CVR), 250 68 Husinec-Řež, Czech Republic; jan.lorincik@cvrez.cz (J.L.); anna.hojna@cvrez.cz (A.H.)

**Keywords:** ferritic–martensitic steel, liquid metal embrittlement (LME), focused ion beam (FIB), electron backscatter diffraction (EBSD)

## Abstract

Liquid lead-bismuth eutectic (LBE) is one of the candidate materials for advanced nuclear systems. The structural materials used in contact with LBE are selected according to the resistance to liquid metal corrosion, irradiation embrittlement, and compatibility with the coolant. However, simultaneous presence of mechanical strain and LBE environment can induce liquid metal embrittlement (LME) in these materials. In this study, a specimen from candidate ferritic-martensitic steel T91 was tested by Constant Extension Rate Tensile (CERT) test exposed to PbBi environment with oxygen concentration 6 × 10^−6^ wt % at 300 °C up to rupture. Post-test examination using scanning electron microscopy (SEM) showed a deep crack indicating features of LME in the plastic strained region of the tested specimen. Further investigations focused on characterization of the fracture path and microstructure determination using focused ion beam (FIB) and energy dispersive X-ray spectrometry/electron backscatter diffraction (EDX/EBSD). This observation revealed that the dominant LME failure mode of the observed crack is translath or transgranular and the crack stopped at the high-angle grain boundary. The role of oxides in the crack initiation is discussed.

## 1. Introduction

Ferritic martensitic steel T91 is one of the potential structural materials for liquid lead or advanced lead–bismuth alloy cooled nuclear reactors (Gen IV reactors) and one of the candidate targets and coolant materials for experimental accelerator driven systems (ADS). The corrosive nature of the Pb and Pb–Bi eutectic (LBE) towards the steel is the cause of technological limits. To prevent the structural materials from corrosion an oxygen control technique [1] is used to form a passivating oxide film on the steel surface thus blocking access of the environment to the metal. However, if a mechanical strain/stress in contact with LBE is applied, it can induce a phenomenon called Liquid Metal Embrittlement (LME). This phenomenon, resulting in crack initiation and sub-critical crack growth leading to premature failure of metals, was observed in T91 steel when tested in LBE over a wide range of applied experimental conditions [2,3,4,5,6,7,8]. The main prerequisites for the occurrence of LME are wetting by the liquid metal together with sufficient level of applied stress, which probably results in localized plastic deformation [2,6,9].

Understanding of the mechanism of LME in T91 is of high importance for a quantitative prediction of the risks of failure due to LME. The complex microstructure of the T91 steel is the limiting factor for clear understanding of the crack initiation and propagation modes and thus for the description of the LME phenomenon occurring during testing of T91 in LBE. It is a matter of debate if the LME-induced cracking mode is a transgranular cleavage-like or boundary cracking (intergranular cracking at martensite lath boundaries and prior-austenite grain boundaries) [10,11].

Recent study investigated the properties of T91 steel tested by Constant Extension Rate Tensile (CERT) in a requested oxygen concentration LBE environment at 300 °C [12]. The fracture path and the morphology of the crack observed in the plastic strain region of T1 specimen tested up to rupture indicated the LME degradation mechanism. It is important to note, that only one crack initiating from the flat surface with indications of LME was found in this test program (nine specimens). In this paper we report on the study of T91-T1 specimen microstructure along the crack induced by LME by means of scanning electron microscopy (SEM) and Electron Back Scattered Diffraction (EBSD) technique.

## 2. Experiments and Methods

In order to provide direct evidence of the mechanism of LME for T91 steel, mechanical testing of a T91 specimen in contact with liquid metal was carried out. The test provided further evidence on the role of the microstructure in LME.

The ferritic–martensitic steel T91 (Grade 91 Class 2/S50460) has nominal composition (wt %) Fe-8.9Cr-0.9Mo-0.4Mn-0.2Si-0.2V and it was produced by Industeel, ArcelorMittal group (Luxembourg). The T91 steel was austenitized at 1150 °C for 15 min and then water cooled down to room temperature. This was followed by an annealing treatment at 770 °C with a holding time of 3 min/mm followed by air cooling. The material after the heat treatment had a fully martensitic structure with a prior austenitic grain size of 20 µm ± 5 µm [13]. The precipitates observed at the prior-austenite grain and sub-grain boundaries are of the M_23_C_6_ type. Inside of the laths of martensite, carbides of M_6_C type and small coherent vanadium carbo-nitrides VN(C) are also present [13].

**Figure 1:**
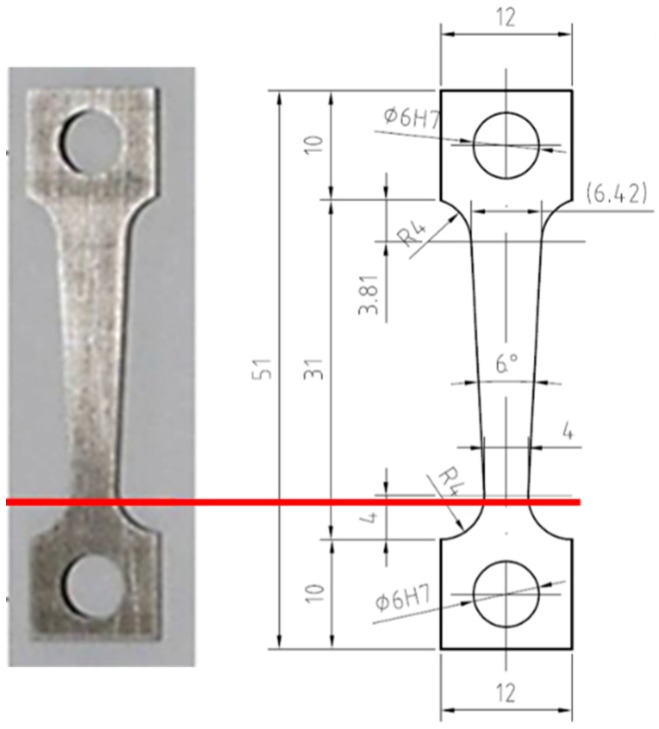
The photo and scheme of the flat tapered specimen used for testing in LBE with main dimensions in mm (red line is a place of rupture).

Constant Extension Rate Tensile (CERT) Test of a flat tapered specimen was used with the aim of determining the threshold stress for crack initiation. The taper creates a variation of stress along the gauge length, which allows identification of the threshold stress for the crack appearance. The flat tapered specimen from T91 steel of the given dimensions (see Figure 1) was manufactured by wire cutting using electrical discharge machining (Emotek, Nové Mesto nad Váhom, Slovakia). One surface of the test specimen was ground to 500-grit finish and the second surface was polished to 1 μm diamond paste, therefore, also the surface finish effect was taken into account. The specimen sides were not treated. The inspection of the specimen surface using SEM (TESCAN, Brno, Czech Republic) revealed flaps and machining grooves as a dominant feature. To check the quality of the prepared surface, surface roughness was measured along the specimen longitudinal axis by means of DektakXT stylus profiler (Bruker, Tucson, AZ, USA). The arithmetical mean roughness (Ra) of the ground surface was 0.047 mm at surface wavelength bandwidth (~0.001 mm, 0.25 mm).

The prepared tapered specimen was tested in static liquid LBE in the CALLISTO cell. This is a tank containing PbBi built on a Zwick/Roell Electromechanical Creep Testing machine (Messphysik, Fürstenfeld, Austria), Kappa 50DS. CALLISTO is based on the 2-tank concepts, where the first tank is for the preparation of the liquid metal (oxygen dosing). The liquid is then transferred to the second tank, containing holders and specimens. The oxygen content was measured in liquid PbBi with oxygen sensors (Bi/Bi_2_O_3_), the requested oxygen concentration achieved was 6 × 10^−6^ wt %. This level is sufficient to develop oxides on the steel [14,15,16]. Flat tapered T91 specimen was monotonic tensile loaded in the CALLISTO cell, at a temperature of 300 °C in PbBi eutectic (LBE) with a strain rate of 1 × 10^−6^/s. More details about the experimental setup and test conditions are given in [12].

After the testing, the specimen was observed and analyzed using dual beam FIB–SEM system LYRA3 GMU (TESCAN, Brno, Czech Republic). The sample surface was observed and documented in secondary electron imaging mode (SEI). The cross-sectional view of the inspected crack was prepared and examined using the FIB milling and polishing procedure with Ga^+^ ions at an accelerating voltage of 30 kV and ion beam currents ~15 nA, ~1 nA, and ~0.2 nA. A protective Pt layer was placed over the area of interest to avoid damaging of the cross-section during ion milling. Subsequent extraction of the lamella for EBSD was done with a standard FIB lift-out technique using an Omniprobe 400 nanomanipulator (Oxford Instruments Nanoanalysis, High Wycombe, UK), the extracted lamella was placed on a transmission electron microscopy (TEM) grid and further thinned and polished in order to prepare a high-quality surface suitable for high resolution EBSD analyses. The crystallographic orientations of the grains located at the sides of the crack were measured using a fast Nordlys Max3 EBSD camera (Oxford Instruments Nanoanalysis, High Wycombe, UK) coupled with an energy dispersive X-ray spectrometer (EDS, Oxford Instruments) allowing simultaneous chemical and microstructural investigation. For the EBSD phase identification, we used the structure of ferrite (iron-bcc). A small step size of 50 nm was used to be able to depict the martensitic laths. The analytical conditions used were 20 kV accelerating voltage, ~3 nA beam current, spot size ~20 nm.

## 3. Results

**Figure 2:**
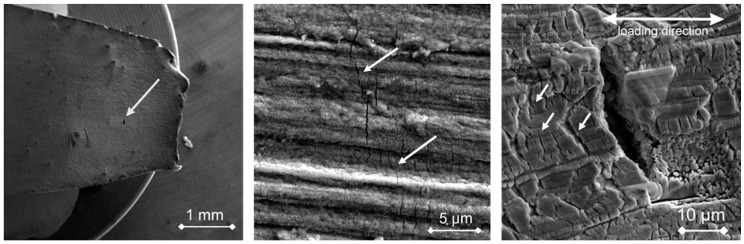
The flat tapered specimen T1 after the CERT test up to rupture, the position of the LME crack in the region of plastic deformation is indicated by arrow (**left**). Slip band cracks are indicated by arrows (**middle**). Detail of the morphology of the large open surface crack selected for further investigation, the crack is oriented ~90° towards the load axis. Note the large number of fine shallow cracks limited to the depth of the oxide layer are indicated by white arrows (**right**).

The CERT test of the investigated specimen (T1) was carried out up to rupture. The maximal stress achieved was 664 MPa (i.e., about the ultimate tensile strength) and plastic strain at minimum diameter reached 4.4%. Two cleavage-like facets initiating from the corners between the ground surface and the sides were found on the fracture surface indicating LME [12]. Several types of cracks were observed on the ground surface of this specimen during the post-test examination (i) slip band cracks, (ii) shallow cracks limited to the oxide layer and most interestingly, (iii) only one perpendicularly oriented and opened crack found in the region of high plastic deformation and maximum stress. Morphology of this crack indicated the possible LME degradation mechanism (see Figure 2).

**Figure 3:**
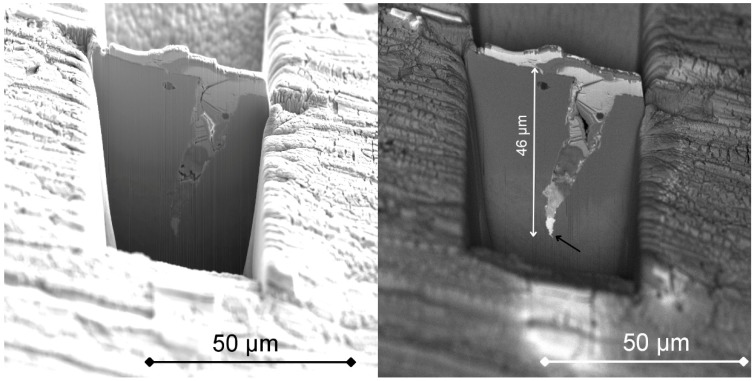
Secondary electron (SE) (**left**) and backscattered electron (BSE) (**right**) images of the investigated crack in the FIB cross-section (backside trench is prepared for further lamella lift-out). The crack is widely opened at the top and filled with liquid metal Pb-Bi on the sides and at the crack tip, as is indicated by the black arrow.

**Figure 4:**
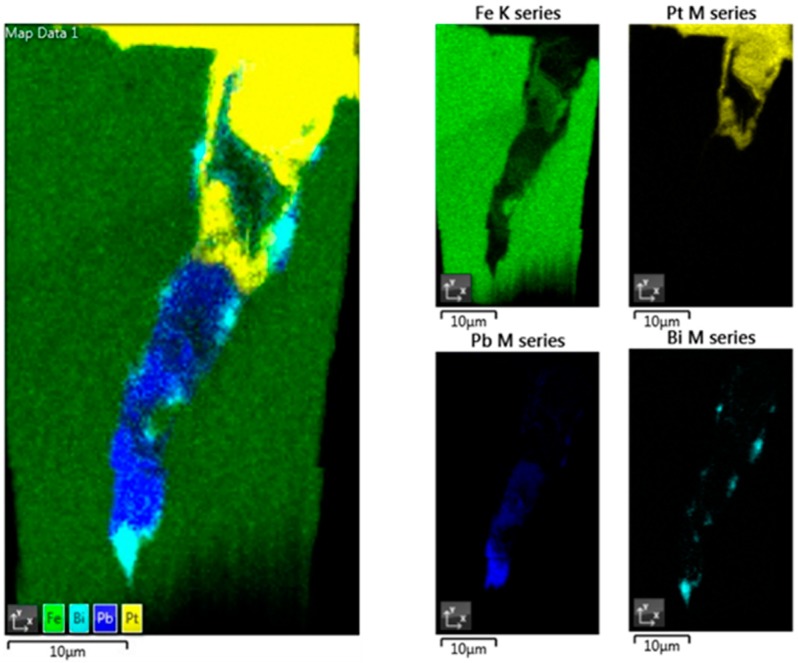
EDS X-ray layered elemental map showing the penetration of LBE into the crack in the T91 specimen and elemental maps for Fe, Pt (coming from the cross-section sample preparation), Pb and Bi of the area around the crack tip.

For investigation of the crack initiation in more detail, an FIB-SEM cross-section following the crack path was prepared. The crack was partly filled by Pt during deposition of the protective layer. Under the protective Pt layer, a complex oxide layer having thickness less than 1 μm can be observed. The oxide layer is formed by an inner spinel FeCr_2_O_4_ and outer magnetite Fe_2_O_3_ structure [17]. The crack tip was reached at a depth of ~46 µm beneath the specimen surface and from the BSE image of the cross-section it is evident that the crack tip is filled with material having high atomic mass (Figure 3). Further EDS analysis of the lift-out lamella confirmed the presence of Pb and Bi only along the crack walls and at the crack tip (Figure 4). No Pb or Bi penetrated into the bulk metal. No significant change in the chemical composition of the ferritic–martensitic matrix can be seen.

**Figure 5:**
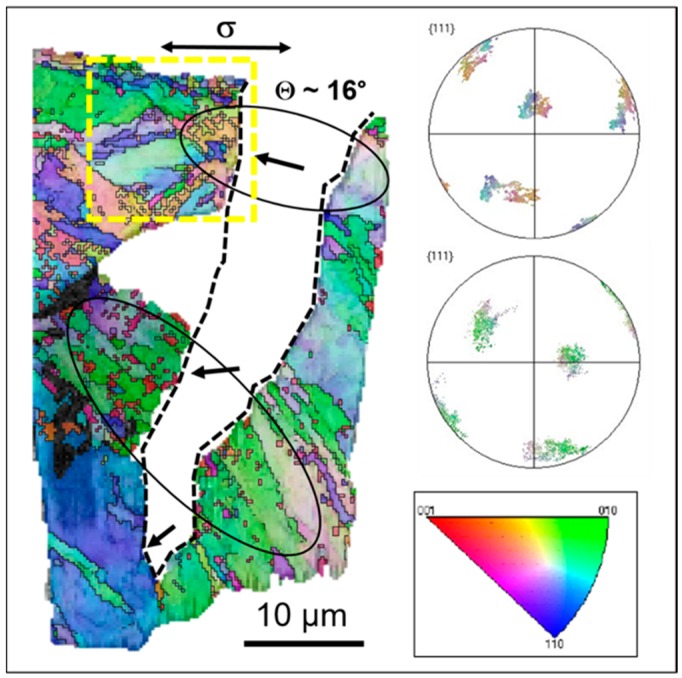
EBSD orientation map (transverse direction (TD) inverse pole figure color coding) exhibiting mainly LME-induced transgranular and translath crack propagation and intergranular cracking towards the tip (marked with black arrows). Pole figures show the orientation of two different families of martensitic laths. The yellow dashed region was investigated further in more detail (see Figure 6).

The EBSD characterization of the microstructure in the vicinity of the crack walls was done simultaneously with the chemical characterization of the lamella by EDS. The EBSD orientation map in Figure 5 confirms, that the LME crack primarily cuts through the mechanically deformed surface layer and then interacts with the microstructure inside formed by lath-like martensite. The crack propagates mainly by transgranular and translath cracking (on a larger scale) without inducing grain refinement or significant microstructure changes and towards the tip it shows the occurrence of intergranular cracking. The crack stops at the high-angle grain boundary. It is interesting to note that the intergranular cracking occurs at the high-angle grain boundary oriented more or less parallel to the crack propagation direction.

**Figure 6:**
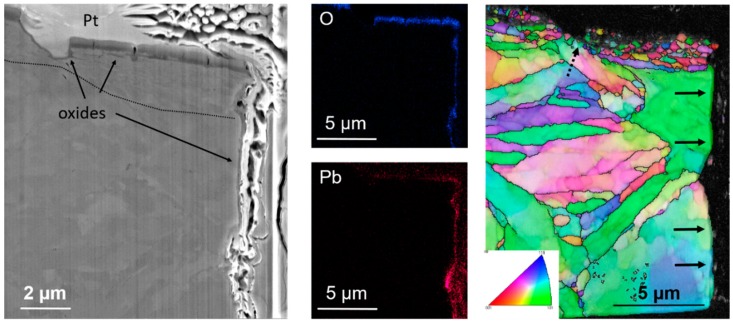
Secondary electron image (**left**) of the upper left part of the crack showing the complex double-oxide layer. Oxide veinlets penetrate into the nanocrystalline subsurface layer, marked with the dashed line. EDS maps of O and Pb illustrate the oxide formation on the surface and walls of the crack on the steel-LBE contact (**middle**). EBSD orientation map (TD inverse pole figure color coding) with high-angle grain boundaries is highlighted. The main crack path is indicated by black arrows. In the area marked with dashed arrows the shallow micro-crack reaches the underlying martensitic laths (**right**).

Further investigations were focused on the upper part of the lamella in order to see the crack initiation region in more detail. The main microstructural feature under the original specimen surface is the deformed fine-grained layer under the metal-oxide interface (Figure 6). This plastically deformed layer was formed during the specimen machining and grinding (500-grid finish) causing shear deformation. It resulted in significant microstructure refinement and dynamic recrystallization, leading to formation of near-surface nanocrystalline deformed layer with a microstructure different from that of the underlying bulk alloy, having the typical ferritic-martensitic lath-like structure.

Figure 6 also shows that there are several micro and nano cracks within the thin protective oxide layer. Oxide veinlets penetrate into the nanocrystalline subsurface layer, marked with the dashed line. From the EDS mapping we can see, that the oxide layer was formed on the surface and also on the walls of the crack, being in contact with the penetrating LBE. There is another shallow crack which stopped in the nanocrystalline layer. The crack deviated by about 45° towards the load axis indicating that the crack propagation is in ductile failure mode.

## 4. Discussion

This work shows new experimental data obtained from a tapered T91 ferritic-martensitic steel specimen tested in LBE under applied stress and strain to initiate liquid metal embrittlement. An advanced FIB-SEM technique was used to prepare a cross-section from the unique crack with indication of LME. The combination of EDS and high-resolution EBSD confirmed that the crack initiation, opening, and propagation was supported by environmentally assisted cracking (EAC) called LME.

It has been shown many times, that to initiate liquid metal environmentally assisted crack with the cleavage-like fracture pattern in T91 in the LBE condition, rupture of the protective oxide layer is needed [2,3,4,5,6,7,8]. Once the protective oxide is broken, wetting together with sufficient stress at the initial micro-crack can result in fast crack growth up to failure regardless of the complex subsurface microstructure, even if grain boundary wetting is thermodynamically preferable than the wetting of the bulk [2]. It is well known that the corrosion resistance of an alloy is attributed to the formation of a protective passive film on its surface. The investigated specimen has a double-layer oxide about ~1 μm thick (see Figure 6). Locally, thicker inner oxide layer zones were observed. In the previous paper [13], ideas of LME crack initiation from the breaking of the outer magnetite under conditions of abrupt tension or cyclic stress was described. Liquid metal can then penetrate through the spinel layer and be in a direct contact with the metal (wetting). The initiated crack can further grow into the metal causing an opening path of liquid metal entry. It seems that the crack development confirmed the idea.

The investigated specimen has a surface affected zone (deformed layer) of ~2–3 μm thick. The EBSD orientation mapping indicated a large number of high-angle grain boundaries which can serve as initiation sites of LME in the case of oxide cracking due to straining in the region of high plastic deformation. These subsurface nanocrystalline grains and elongated micro-bands contain a high density of grain boundaries providing a large number of diffusion pathways for Cr and O, which may contribute to the faster inward oxidation of the steel and de-lamination of the fine-grained layer. This can have a substantial effect on the behavior of materials in any environment under introduced stress. In the case of LBE, the applied stress and strain might cause damage of the oxide, preventing wetting and this could be the initiation site of LME.

The bulk microstructure of T91 steel is rather complex, consisting of prior-austenite grains containing several packets of laths, formed as a result of martensitic transformation during the manufacturing of the steel. The presence of different types of grain boundaries (i.e., low-angle and high-angle) predetermines the T91 steel to be subjected to multiple failure modes when the fracture is induced by LME. In previous studies, fracture surfaces or large cracks were observed by EBSD to address the issue of the LME failure mode [9,10,11]. Martin et al. described the observed intergranular cracking at martensite lath boundaries and prior-austenite grain boundaries as the main LME mechanism in T91 steel [10]. On the contrary, the microstructural observations of Gong et al. on the fatigue crack propagation modes revealed that the dominant LME mode was transgranular or translath, rather than intergranular or interlath cracking. The intergranular or interlath decohesion was found to be orientation dependent [9]. Our observation has shown that the lath boundaries or the prior-austenite grain boundaries do not necessarily act as preferential paths for LME crack initiation and the observed cracking close to the original specimen surface has mostly transgranular features. The LME crack propagation direction is more or less perpendicular to the loading axis, which is in good agreement with the cleavage-like fracture mode. No induced grain refinement was observed along the crack path, which indicates that the plastic deformation around the crack tip necessary for the presence of LME was low. This fact is fully in agreement with a previous observation [7] showing that EAC/LME crack was propagated by a fracture mechanics specimen at high load without buckling on the specimen sides. 

## 5. Conclusions

The fracture mode of the surface crack in T91 ferritic–martensitic steel tested by CERT test in contact with lead–bismuth eutectic liquid metal at 300 °C was investigated by determining the microstructure along the fracture path. To examine the role of LME on the cracking, scanning electron microscopy (SEM) with focused ion beam (FIB) and simultaneous EDS + EBSD analytical techniques were used. The BSE imaging and EDS mapping confirmed that the crack initiation and growth was supported by environmentally assisted cracking called LME and that Pb-Bi remained on the crack surface only. EBSD orientation maps showed that the crack propagates mainly through the sub-grain and lath boundaries and stopped on the high-angle grain boundary. A deformed nano-crystalline layer developed under the surface serving as the initiation site of LME in synergy with protective oxide cracking.
